# Treating Diabetes with Exercise – Focus on the Microvasculature

**Published:** 2013-11-16

**Authors:** CM Kolka

**Affiliations:** Department of Biomedical Sciences, Diabetes and Obesity Research Institute, Cedars-Sinai Medical Center, CA, USA

**Keywords:** Exercise, Diabetes, Insulin, Muscle, Vasculature, Blood vessels

## Abstract

The rising incidence of diabetes and the associated metabolic diseases including obesity, cardiovascular disease and hypertension have led to investigation of a number of drugs to treat these diseases. However, lifestyle interventions including diet and exercise remain the first line of defense. The benefits of exercise are typically presented in terms of weight loss, improved body composition and reduced fat mass, but exercise can have many other beneficial effects. Acute effects of exercise include major changes in blood flow through active muscle, an active hyperemia that increases the delivery of oxygen to the working muscle fibers. Longer term exercise training can affect the vasculature, improving endothelial health and possibly basal metabolic rates. Further, insulin sensitivity is improved both acutely after a single bout of exercise and shows chronic effects with exercise training, effectively reducing diabetes risk. Exercise-mediated improvements in endothelial function may also reduce complications associated with both diabetes and other metabolic disease. Thus, while drugs to improve microvascular function in diabetes continue to be investigated, exercise can also provide many similar benefits on endothelial function and should remain the first prescription when treating insulin resistance and diabetes. This review will investigate the effects of exercise on the blood vessel and the potential benefits of exercise on cardiovascular disease and diabetes.

The prevalence of diabetes has been increasing steadily in the United States and in many parts of the world. In 2010, 25.8 million individuals in the United States were diagnosed with diabetes, a figure almost double that of ten years previously [1]. Diabetes frequently occurs with other diseases, including dislipidemia, hypertension, cardiovascular disease and obesity. Common complications of diabetes include heart disease, blindness, kidney disease and peripheral neuropathy, often leading to amputation. People with type 2 diabetes are typically sedentary, overweight, and have decreased physical fitness [2], and the Center for Disease Control and Prevention and the American Heart Association consider lack of physical activity as a risk factor for heart disease [3].

Currently the first treatment prescribed for type 2 diabetes is lifestyle modification, including diet and exercise, though drugs are used when lifestyle changes are not sufficient. Weight loss is a primary recommendation in overweight or obese patients, particularly those with type 2 diabetes, and can show many short term benefits, such as improvements in glycemic control, reduction of cardiovascular risk factors, and resolution of coexisting illnesses. Lifestyle intervention alone can cause significant weight loss and at least a partial remission of diabetes [4].

The contribution of exercise to weight loss specifically is controversial, and studies have shown only an incrementally greater weight loss by exercise and diet over diet interventions alone. However, weight loss is not required for resolution of diabetes, and some drugs increase body weight while improving insulin sensitivity, such as the thiazolidinediones [5]. Thus obesity and increased fat mass are not always directly linked to diabetes: while the majority of those with type 2 diabetes are overweight, a large proportion of obese individuals are not diabetic. Yet obesity is a major risk factor for developing diabetes. The location of fat tissue is a major determinant of insulin resistance, as visceral fat is associated with insulin resistance [6], and subcutaneous fat deposition confers a protective effect against diabetes [7]. Obesity and increased fat mass can determine diabetes and cardiovascular risk [8], thus an intervention to reduce body fat will also reduce diabetes risk. Exercise can reduce fat mass independently of changes in total body weight [9].

Exercise is also associated with significant improvements in other aspects of disease, such as the reduction of complications, associated metabolic diseases, and other risk factors [9]. The metabolic syndrome, typified by high blood pressure, high triglyceride levels, low HDL-cholesterol levels, high fasting glucose, and central obesity, is recognized to predispose individuals to the development of diabetes and atherosclerosis. Interestingly, most of the criteria of the metabolic syndrome pertain to blood measurements, and can therefore affect blood vessels. Further, many of the complications of diabetes, including retinopathy, kidney disease and peripheral neuropathy, also have a vascular basis. In their review, Joyner and Green note that exercise is much more protective against cardiovascular disease than would be expected based on changes in traditional risk factors, including BMI, blood lipids and blood pressure [10]. They suggest a vicious cycle between autonomic dysfunction and endothelial dysfunction leading to cardiovascular disease, which can be prevented by exercise [10]. Here, the role of the endothelium and microvasculature in exercise and diabetes is reviewed.

## Exercise as Treatment for Diabetes

Type 2 diabetes occurs when the body cannot maintain normal blood sugar levels. In the early stages of the disease insulin is unable to stimulate glucose storage in appropriate tissues. To compensate, the pancreas releases more hormone, but eventually fatigues, leading to insulin deficiency. Skeletal muscle [11] and liver insulin resistance [12] have both been proposed as the primary defect in type 2 diabetes, and the implication is that cellular insulin resistance is the major issue. There have been many studies investigating insulin signaling cascades in skeletal muscle [13–15] and a variety of other cell types [16,17], and both receptor defects and post-receptor signaling defects have been observed [18] yet insulin must get to the cells before it can engage the receptors, and relies on a functioning microvasculature for access. In the vasculature both endothelial [19–21] and vascular smooth muscle cells [22] have shown insulin signaling defects, and functional vascular impairments are also evident. In healthy individuals insulin signaling in the endothelium can increase perfusion of muscle, improving the delivery of nutrients and hormones to muscle [23]. Insulin sensitivity is strongly related to the ability of insulin to access muscle: this access is impaired in cases of both acute and chronic insulin resistance [24,25], and is likely due to impaired endothelial function. Endothelial dysfunction is evident in diabetes and even pre-diabetes [26,27], and men with diabetes have both impaired endothelium-dependent and endothelium-independent vasodilation [28]. Further, endothelial dysfunction is associated with a family history of diabetes [29], even in otherwise healthy individuals.

## Vascular Effects of Exercise

**Figure 1:**
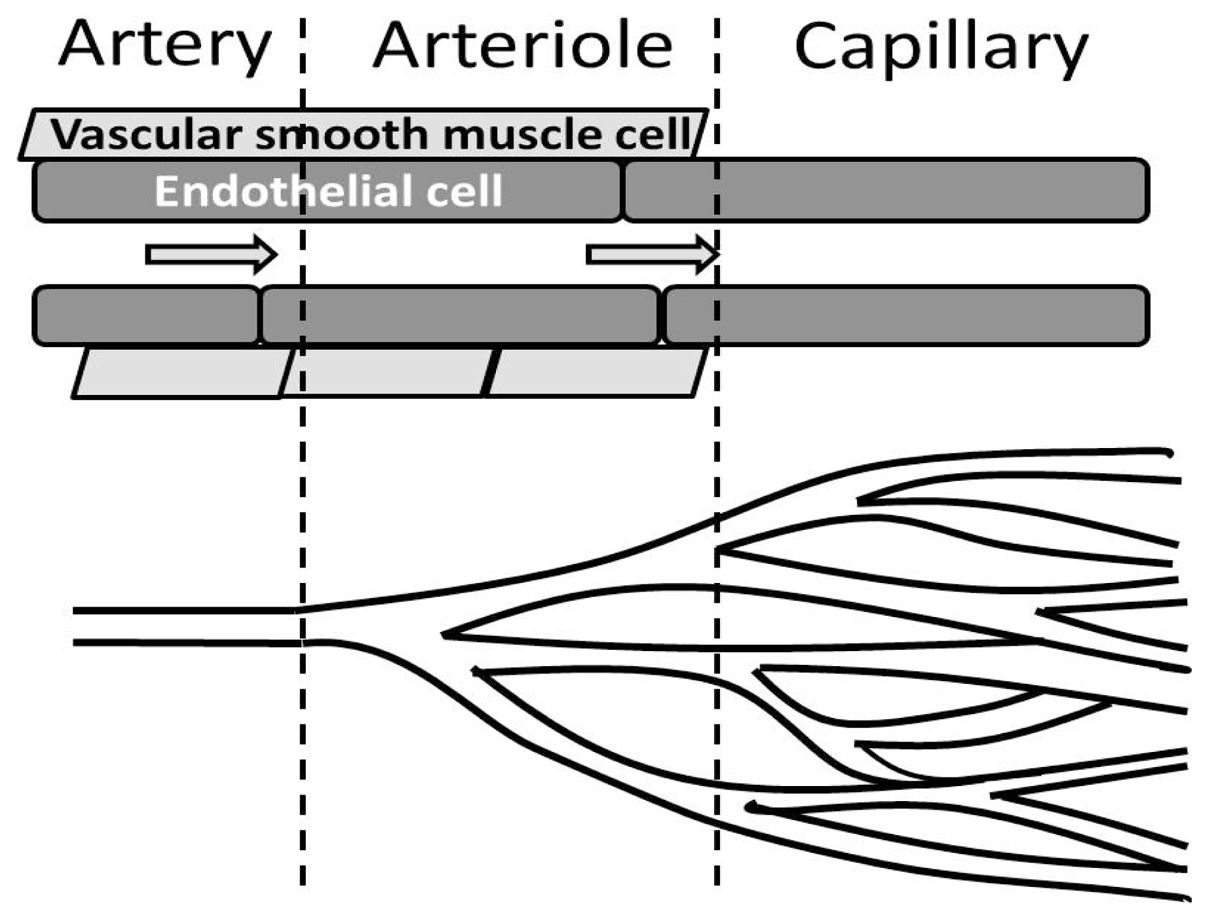
Structural differences between artery, arteriole and capillary. No vascular smooth muscle is located on the capillary; therefore flow through capillaires is modified by pre-capillary arterioles. Cessation of flow through arterioles will prevent flow through a portion of the muscle.

Muscle is the focal point during exercise, but is also a major metabolic organ, and the primary site for insulin-mediated glucose metabolism. Incremental changes in exercise intensity are matched by the amplitude increase in blood flow specifically to muscle, with only small effects or even decreases observed in other tissues [30]. This increase in blood flow to active tissue is termed active hyperemia, or functional hyperemia. Bulk blood flow to muscle can change significantly, particularly with exercise [31], but the distribution of blood through the muscle can be altered even with no changes in total flow [32]. Light exercise in humans causes a short term increase in forearm blood flow within 5 seconds of contraction. However, exercise also has a major effect also on microvascular blood volume, even when blood flow effects had returned to normal [32]. At rest, a low proportion of capillaries are exposed to blood flow at one time, with a rapid increase in the number of perfused capillaries after exercise [31], thus increasing functional capillary density. The microvasculature in the working muscle is selectively recruited [33], and those areas with lowest perfusion in the working muscle are recruited first [34]. Different muscle fibers serve different roles in the body, with highly oxidative muscle being engaged during exercise, and glycolytic muscle fibers performing more of a postural or structural role. Blood flow is closely coupled with the contraction of the muscle fibers [35], such that the magnitude of flow in each muscle fiber type reflects activity and oxidative metabolism of the muscle[36]. The mediators responsible for controlling muscle blood flow during exercise can arise from the muscle, nerves and the endothelium of blood vessels [34,37]. Vascular smooth muscle cells are located around the arterioles and some venules, and can constrict to change blood flow patterns, while capillaries do not typically contribute to blood flow changes [30] (Figure 1). Blood flow through capillaries is controlled upstream by small arterioles at rest, and the rapid recruitment of unperfused capillaries by exercise could suggest that nerves are responsible for this action [34]. The sympathetic nervous system is mainly responsible for the vasoconstrictor responses, and as the arterioles and larger vessels are innervated [38] the majority of sympathetic nervous system activity is localized to that area of the vascular tree. Physical exercise can enhance sympathetic nerve activity [39] to maintain arterial pressure, and may be involved in maintaining exercise tolerance, as reviewed by Thomas and Segal [38]. More recent studies have suggested organ specific differences in sympathetic nervous system activity with weight loss [40]. While exercise training has short term effects to improve sympathetic response [39], addition of aerobic exercise to a weight loss program did not augment any sympathetic changes [41], thus exercise training effects on the sympathetic nervous system may be due purely to a reduction in body weight. We suggest that short term effects of exercise on the sympathetic response are evident, but the contribution of the sympathetic nervous system activity to the beneficial effects of a long-term exercise intervention is uncertain, and instead functional improvement of the blood vessels remains a likely contributor to the benefits of exercise.

Insulin relies on endothelium-dependent vasodilation to enhance perfusion, thus endothelial dysfunction reduces insulin-mediated increases in muscle perfusion, which can contribute to the metabolic deficit in diabetes. As exercise-mediated changes in perfusion are typically endothelium-independent, exercise is still able to recruit capillaries and thus increase muscle perfusion in obesity and type 2 diabetes, even in the face of endothelial dysfunction. Numerous studies have now shown that while insulin’s vascular effects may be blocked in diabetes, exercise still maintains its ability to increase the distribution of blood flow through muscle [42]. While physical inactivity is associated with impaired microvascular function [43] training programs improve endothelial function [44]. However, while uncomplicated type 2 diabetic patients show normal capillary recruitment responses to exercise, in type 2 diabetic patients that also have microvascular complications this response is impaired [45], likely due to a functional impairment of blood vessels rather than morphological changes. The reduced exercise capacity observed in type 2 diabetics can be overcome with an exercise training program, though even when matched for physical activity and weight, diabetic patients have decreased physical fitness [2].

Nitric oxide (NO) is the main vasodilator from the endothelium specifically involved in blood flow and blood distribution, and while reduction in nitric oxide synthesis lowered total blood flow, exercise-mediated capillary recruitment was not affected [46]. In fact, inhibition of NO formation enhances both resting and exercise-mediated muscle oxygen uptake [47]; despite a reduction in total flow, microvascular flow was not affected, suggesting that NO is not involved in the vascular response to exercise. However, other studies have shown that exercise training required nitric oxide for improvements in flow-induced dilation [44]. It is therefore possible that while NO is not involved in the acute response to exercise, exercise training restores general endothelial health, as evidenced by a restored endothelium-dependent vasodilation in response to flow. Thus, as well as the acute effects of exercise which may be independent of NO, an exercise regimen may improve endothelial function.

## Metabolic Effects of Exercise

**Figure 2:**
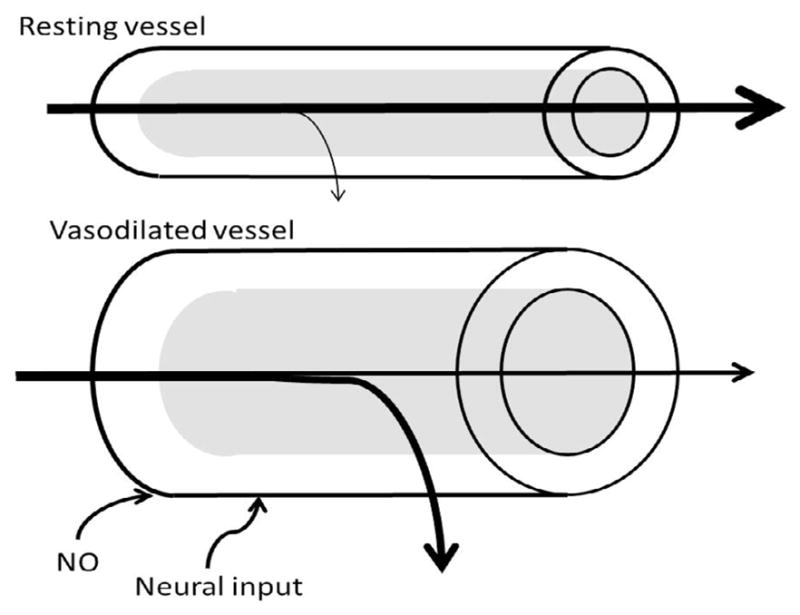
Vasodilation affects delivery, and thus metabolism. The rate of transfer across the endothelium is dependent on surface area, permeability of the endothelium, diffusion distance, and concentration difference (Fick’s first law of diffusion). Vasodilation increases surface area in arterioles for exchange, but will also recruit downstream capillaries, which will reduce diffusion distance and increase surface area for exchange. Working muscle increases oxygen utilization, increasing the concentration difference from the blood vessel to the tissue.

The distribution of blood through muscle increases the capacity for nutrient exchange. In exercise the primary purpose of functional hyperemiais for oxygen delivery, as the oxygen required by exercising muscle is much higher than resting muscle (reviewed in [37]). Recruitment of capillaries can decrease the velocity of blood flow by increasing the cross-sectional area of the capillary bed and the time available for exchange. Recruitment also increases surface area for exchange and decreases perfusion distances to promote oxygen delivery to tissues with exercise [34] (Figure 2). While in exercise the main metabolite required at the working muscle is oxygen, distribution of other nutrients can also be affected, including glucose, fats, other hormones and cytokines. Muscle metabolism can therefore be altered by perfusion of the tissue [48,49]. While there can be regulated transport of certain larger hormones across the vasculature [50,51], smaller molecules can diffuse across the endothelium easily, possibly making muscle perfusion a more important player in the delivery of glucose and oxygen to the tissue.

Skeletal muscle is the main site of basal glucose uptake, and is the tissue most associated with exercise; therefore the effect of exercise would likely be localized in muscle. A single bout of exercise in sedentary men increases glucose uptake and glucose effectiveness, and it was suggested that the increased blood flow and distribution enhanced glucose delivery to the tissue [52]. Capillary recruitment with exercise contributes to glucose uptake, but NO is not required for exercise mediated capillary recruitment [46]. Instead, NO augments glucose uptake in high intensity exercise [46], but not low intensity exercise, and may be involved in a partitioning of fuel utilization [53]. Longer term, mild exercise training improves glucose disposal, even with no change in body composition [54]. This sustained effect was independent of the metabolic benefits of a single bout of exercise. Changes of insulin-specific glucose transporter expression have been detected after exercise training [55,56], as have changes in DNA methylation [57], but it is also possible that general improvements in endothelial function increase delivery, and thus metabolism, of glucose.

Fat deposition in muscle is often thought to be associated with insulin resistance [58,59], and selective reduction of intramyocellular lipid restores normal insulin signaling, reverting to a healthy metabolic state [60]. Thus, rather than intramuscular or total body fat, intramyocellular fat is related to muscle insulin resistance. However a paradox is noted when athletes are considered, as they often have very high levels of intramyocellular lipid, yet high insulin sensitivity [61]. Intramyocellular lipid content is increased after exercise intervention and diet change, coinciding with an increase in insulin sensitivity, suggesting that intramyocellular lipid content may not directly impair cellular insulin sensitivity [62]. Exercise can prevent lipid-induced insulin resistance [63], and the form the lipid is stored in may contribute to insulin resistance, asceramide or diacylglycerol [64–66] are more detrimental to cellular insulin action than triglyceride. Another component of the divergent effects of intramyocellular lipid on insulin action could be the site of storage of excess fat. Lipid droplets within the muscle cell may regulate insulin action [67] and possibly mitochondria such that lipid-droplet derived fats can be used as fuel by exercising muscle [68]. In contrast, nutrient overload can alter the lipid droplet coat proteins and change the interaction of the lipid droplet with other organelles, causing inflammation and oxidative stress. Thus, while fat deposition in muscle may not directly affect vascular function, the resulting inflammation [69] and oxidative stress [70] from intramyocellular lipid can lead to endothelial dysfunction. Further, fat deposition in endothelial cells has not been directly measured, and may occur in a similar fashion as in muscle and directly affect vascular function.

Muscle is composed of oxidative and glycolytic fiber types, with oxidative fibers typically having more mitochondria, and being actively recruited during exercise. The density of capillaries is greater in oxidative muscle, reduced oxidative activity in type 2 diabetic patients is most likely due to a reduction in slow oxidative fibers [71]. The decrease in oxidative activity and increase in glycolytic activity in these patients was closely linked to the fraction of each fiber type present in muscle, suggesting that type 2 diabetic patients show both changes in fiber composition and fiber-specific metabolism. Mitochondrial dysfunction has been proposed to be both a cause [72] and a consequence [73] of insulin resistance, and may contribute to endothelial dysfunction [74]. If oxygen delivery is a component of mitochondrial health and biogenesis, it is possible that impaired perfusion may contribute to fiber type switching, where an oxidative fiber, which is typically highly vascularized and contains mitochondria, switches to a glycolytic fiber with less vascularity and mitochondria. As exercise can improve oxidative capacity, increase mitochondria content [75], and also increase muscle perfusion [31,32,34,45,76], the relationship between muscle perfusion, fiber type and mitochondrial function needs to be clarified.

Exercise training may or may not have effects on basal metabolic rate. In older adults, 26 weeks of training increased resting energy expenditure, and also improved lipid oxidation rates [77]. Habitually active women were also found to have a higher resting metabolic rate than matched sedentary controls, associated with lower body fat levels [78]. However, there are a variety of studies that show no effect of exercise intervention on basal metabolic rate, such as one that used a 26 week training program investigating a mix of aerobic and resistance training [79]. These individuals were previously sedentary, and had a history of type 2 diabetes. Many aerobic training studies fail to show an improvement in resting metabolic rate, and Jennings et al. [79] note that resistance training intensity or frequency may increase fat-free mass, which is the primary cause of resting metabolic rate changes [80]. Thus, the lack of improvement in basal metabolic rate may be due to no significant change in fat free mass [79] or the reduced exercise capacity of diabetic patients [2].

## Treating Metabolic Disease

Aside from improvements in endothelial function, exercise can also affect metabolism, and this can be exploited in metabolic disease such as diabetes. Systemic vascular improvement can also improve insulin sensitivity [81], so targeting the endothelium in diabetes is a valid option for treating metabolic disease [82]. The relationship between vascular action and metabolism has been previously reviewed [83], and impaired vascular function has been implicated as the link between obesity and diabetes [84]. Essentially, without appropriate blood flow, distribution of blood through tissues, or transport from the vessels, metabolic function is limited due to reduced nutrient and hormone availability.

Exercise training improves insulin sensitivity [54] and while this can be due to an increase in insulin specific glucose transporters after exercise [85] blood flow distribution changes may also indirectly improve metabolism. In rodent models of obesity that show a failure of insulin to increase muscle perfusion, muscle contraction can still cause capillary recruitment and glucose uptake [42]. Insulin and exercise have an additive effect on glucose uptake in muscle, and the authors discuss the potential contribution of blood flow and capillary surface area to their results [86]. In obese patients the defect in insulin-mediated skeletal muscle perfusion was restored by exercise, yet cellular insulin resistance was still evident [87]. Thus while exercise does increase the effect of insulin on glucose metabolism in both lean and obese individuals, it does not normalize the cellular deficit due to obesity. The increased insulin-mediated glucose uptake observed with exercise training is likely due to improved hemodynamic effects in muscle [76].

## Complications

Insulin resistance per se may underlie the development of other aspects of the metabolic syndrome [81] and many of these can have a vascular basis. Targeting endothelial dysfunction is therefore a viable treatment for preventing vascular complications associated with diabetes [70]. The vascular component of exercise may well be linked to the reduction of diabetic complication such as retinopathy, peripheral neuropathy and nephropathy, as there is a vascular basis to many of these complications. The endothelium has been implicated in diabetic nephropathy [88], and the blood vessels formed in response to reduced perfusion in retinopathy show abnormal structure and function [89]. Endothelial dysfunction is evident in hypertension and cardiovascular disease, and is also noted in many cardiovascular risk factors, including abnormal blood lipid levels, and hyperglycemia. Treatment of those risk factors typically restores endothelial function. Therefore systemic vascular protection has been proposed as a treatment for type 2 diabetes, that would prevent complications, but also improve insulin sensitivity [81]. Physical exercise is anti-atherogenic [90], but also confers general vascular protection, and as such could prevent many of the complications associated with diabetes.

## Negative or Neutral Outcomes of Exercise

Lifestyle interventions such as diet and exercise are the first recommendation for treatment of diabetes and obesity, yet drug treatment is a very common therapy. While diabetic patients have defects in exercise capacity [2], this can be improved by either exercise training, or agents that improve insulin sensitivity. Certain hormones can be upregulated in metabolic disease, such as endothelin-1 in hypertension, and excessive levels of endothelin-1 can reduce aerobic capacity of muscle, and impair metabolism [91], most likely through impaired blood flow. Investigations are ongoing into certain drugs that are designed to mimic exercise. For example, sildenafil [92] and AICAR [93] have been shown to increase peripheral microcirculation. However, there can be adverse effects of various drugs in combination with exercise too. For example, rosiglitazone usage may improve exercise capacity, but may contribute to heart failure [94].

The Look AHEAD study shows diet and exercise as part of an intensive lifestyle intervention have no significant effect to lower cardiovascular events in overweight or obese individuals, which could suggest that exercise has no long term cardiovascular benefit [4], and complete remission of type 2 diabetes is rare [95]. However, the control group in this study was assigned to diabetes support and education, and no measure of physical activity or diet changes was performed in this group. Thus while 6 kg weight loss was achieved by diet and exercise after nearly ten years, the control group also showed weight loss of 4 kg [4]. The use of drugs in the Look AHEAD study may also explain the apparent lack of improvement in cardiovascular outcomes with lifestyle intervention [4], based on potential drug interactions listed above. However, the same study did show partial remission of type 2 diabetes [95], and noted that improvements in glycemic control by exercise were dependent on the blood glucose level prior to beginning the intervention [96]. A similar study investigated lifestyle intervention in overweight people with impaired glucose tolerance, and similarly showed no effect of intervention to decrease cardiovascular morbidity after 10 years. However this study showed a decrease in the incidence of type 2 diabetes in the lifestyle intervention group, thus exercise and diet was able to reduce type 2 diabetes incidence [97]. Therefore, exercise should be an early intervention to prevent type 2 diabetes and obesity, as it is more effective after a shorter duration of diabetes [96], and can prevent at-risk individuals from progressing to type 2 diabetes [97]. Further, short term exercise interventions have caused weight loss, restored insulin sensitivity, as well as improved cardiometabolic risk factors [98]. Therefore, exercise is an effective intervention early in the progression of disease, and has some benefits even in established diabetes. Further, the lifestyle intervention has documented improvements on other quality of life measures, including sexual functioning in women and obstructive sleep apnea, likely through weight loss.

## Perspectives

Exercise is an important part of a healthy lifestyle, particularly as part of disease prevention rather than cure. Aerobic activity is recommended by the American Heart Association and the American College of Sports Medicine to promote and maintain health, particularly in respect to cardiovascular disease, stroke, hypertension, type 2 diabetes, obesity, and other common diseases [3]. Further, incorporation of resistance training may have additional benefits [80]. Exercise has reduced efficiency in established type 2 diabetes [2] and the duration of diabetes may also be responsible for the lack of improvement in resting energy expenditure in diabetic patients [79]. The clinical applicability of exercise in established diabetes will still improve factors discussed above such as improving atherosclerosis [90] and insulin sensitivity [54]. In spite of reported negative results [4], exercise may also improve cardiovascular risk factors, and prevent the progression to diabetes [97]. Early adoption of an exercise regimen will therefore provide best results in cardiovascular and metabolic outcomes.

## Conclusion

Due to the rising incidence of diabetes, and the associated metabolic diseases such as obesity, cardiovascular disease and hypertension, lifestyle interventions including diet and exercise are the first line of defense. The benefits are typically thought of in terms of weight loss, improved body composition and reduced fat mass, but exercise can have many other beneficial effects independent of this. Exercise can affect the vasculature, improving endothelial health. Further, insulin sensitivity is improved, and the treatment of endothelial dysfunction may also reduce complications associated with both diabetes and other metabolic disease. While the use of drugs to improve microvascular function in diabetes has previously been reviewed [83], exercise can also provide many of the same benefits on endothelial function, and should remain an early intervention and the first prescription in combination with diet when treating insulin resistance and diabetes.

## Abbreviation

NO: Nitric Oxide
